# Impact and cost-effectiveness evaluation of a community-based rehabilitation intervention on quality of life among Chinese adults with hearing loss: study protocol for a randomized controlled trial

**DOI:** 10.1186/s13063-021-05228-2

**Published:** 2021-04-07

**Authors:** Xin Ye, Dawei Zhu, Siyuan Chen, Xuefeng Shi, Rui Gong, Juncheng Wang, Huibin Zuo, Mei Zhang, Ping He

**Affiliations:** 1 School of Public Health, Peking University, Beijing, China; 2 China Center for Health Development Studies, Peking University, Beijing, China; 3 School of Management, Beijing University of Chinese Medicine, Beijing, China; 4 National Institute of Chinese Medicine Department and Strategy, Beijing University of Chinese Medicine, Beijing, China; 5 China Rehabilitation Research Center for Hearing and Speech Impairment, Beijing, China; 6 Linyi Center for Disease Control and Prevention, Linyi, Shandong China; 7 Linyi Rehabilitation Hospital, Linyi, Shandong China

**Keywords:** Community-based rehabilitation, Hearing loss, Randomized controlled trial, Study protocol

## Abstract

**Background:**

Hearing loss is quite prevalent and can be related to people’s quality of life. To our knowledge, there are limited studies assessing the efficacy of hearing interventions on quality of life in adults. Therefore, we aim to conduct a randomized controlled trial (RCT) to determine the impact and cost-effectiveness of community-based hearing rehabilitation on quality of life among Chinese adults with hearing loss.

**Methods/design:**

In this two-arm feasibility study, participants aged 16 and above with some degree of hearing loss (*n* = 464) will be recruited from Linyi City, Shandong Province. They are randomly assigned to the treatment group or the control group. Those in the treatment group are prescribed with hearing aids, while those in the control group receive no intervention. Reinstruction in use of devices is provided for the treatment group during booster visits held 12 months post-randomization or unscheduled interim visits when necessary. Data are collected at baseline and the follow-up 20 months later. The primary outcome is changes in quality of life over a 20-month study period. Secondary outcomes include sub-dimensions in quality of life, physical functioning, chronic diseases, cognitive function, depression, social support, hospitalizations, falls, and healthcare costs. Finally, we will evaluate whether hearing aids intervention is cost-effective to apply in a large scale.

**Discussion:**

The trial is designed to evaluate the impact and cost-effectiveness of a community-based rehabilitation intervention on quality of life among Chinese adults with hearing loss. We hope that it would help improve the well-being for Chinese adults and provide references in policy and practice for China and other countries.

**Trial registration:**

Chinese Clinical Trial Registry ChiCTR1900024739. Registered on 26 July 2019.

**Supplementary Information:**

The online version contains supplementary material available at 10.1186/s13063-021-05228-2.

## Background

Hearing is one of the basic means of perception and communication. With the global aging, hearing loss is the most common sensory dysfunction and is becoming an increasingly serious public health issue [1]. It is said that more than 90% of the hearing loss is related to aging [2], most of which is irreversible [3]. In the USA, nearly two thirds of people over age 70 had hearing loss in 2015 [4]. In China, according to the Second National Sample Survey on Disability in 2006, the prevalence rate of hearing disability (mild or above) in older adults over 60 years old was about 11% [5], ranking the highest among six categories of disability (hearing, visual, language, physical, intellectual, and mental disabilities) [6].

Hearing loss can be associated with a series of health problems, such as poor physical and mental health [7, 8]. Empirical studies have shown that hearing loss is related to the decline in quality of life, presenting as more comorbid chronic diseases [9]; impaired physical functioning [3, 10]; more depressive symptoms such as sadness, despair, helplessness [9]; and accelerated cognitive decline [9]. The underlying mechanism may be that hearing loss impedes information exchange and social participation, which further impairs active physical functioning, increases psychological burden, and is associated with poor health [3, 10].

Although there is a strong correlation between hearing loss and health, it has not received enough attention from the public. First, hearing loss usually has a slow onset and gets worse progressively, which is difficult to detect in time unless by audiometry tests [11]. Our previous study proved that nearly half of the hearing-impaired people did not find themselves suffering from hearing loss or were not sure when the hearing loss occurred [12]. Second, a large number of people regard hearing loss as a natural aging process that can be disregarded [13]. What is more, the huge costs of hearing treatment like wearing hearing aids undoubtedly prevents the accessibility and utilization of rehabilitation services [14]. All these factors contribute to hearing loss becoming a widespread and undertreated health problem [13].

### The intervention of hearing aids use

The use of hearing aids is a main rehabilitation intervention for people with hearing loss [15]. One study investigating the predictors of rehabilitation intervention decisions in hearing-impaired middle-age and older adults found that hearing aids are more likely to be their first choice under most circumstances [16]. People who wear hearing aids have higher quality of life than those who do not, as evidenced by improved social skills, better self-care and mobility, lower levels of depression, and better overall health [17, 18]. But the accessibility and utilization rate of hearing aids are quite low [14]. Community-based surveys in developed countries have demonstrated that the use of hearing aids for older adults with hearing loss was around 10 to 20% from 1997 to 2005 [19–21]. Studies on hearing aids are scarce in developing countries [22]. Our study conducted in four provinces of China found that only 6.5% of the older adults with hearing loss had hearing aids in 2014 to 2015 [23].

Given the high prevalence of hearing loss and low accessibility of hearing aids, it is imperative to conduct high-quality studies to figure out the efficacy of wearing hearing aids. Systematic reviews on hearing and quality of life have found that a majority of studies were cross-sectional and only a few were conducted in developing countries [24]. Although there is a general consensus that hearing aids are beneficial for people with hearing loss, such as improving social function in Short Form 36 Health Survey (SF-36) [25], reducing risks of anxiety and depression in EuroQoL 5-Dimension (EQ-5D) [26], some studies suggest no evidence linking hearing aids use to improved quality of life [27, 28]. Besides, no randomized controlled trials (RCTs), to our knowledge, have studied the effects of hearing aids on exact health-related variables like depression, cognitive function, or service utilization in developing nations. Therefore, we supplemented these variables in our RCT as secondary outcomes to present more comprehensive quality of life outcomes, so as to add more compelling clinical evidence whether wearing hearing aids is cost-effective in developing countries like China.

In this protocol, we described the design of a randomized controlled trial to assess the impact and cost-effectiveness of hearing aids intervention on 20-month changes in quality of life. The study is expected to involve 464 Chinese adults aged 16 and older who have some degree of hearing loss and are required to wear a hearing aid by otologists. They are randomly assigned to the treatment group of wearing hearing aids or the control group with no intervention to determine the efficacy of hearing aids in improving the quality of life and to move toward early prevention and treatment of hearing loss.

### Objectives

The primary objective is to determine whether hearing aids intervention is effective in improving the quality of life in adults with hearing loss. The secondary objectives are to determine whether hearing aids treatment is cost-effective, including improving the health-related quality of life, decreasing inpatient and outpatient visits, and recovering productivity.

## Methods/design

### Study design, participants, and setting

**Fig. 1:**
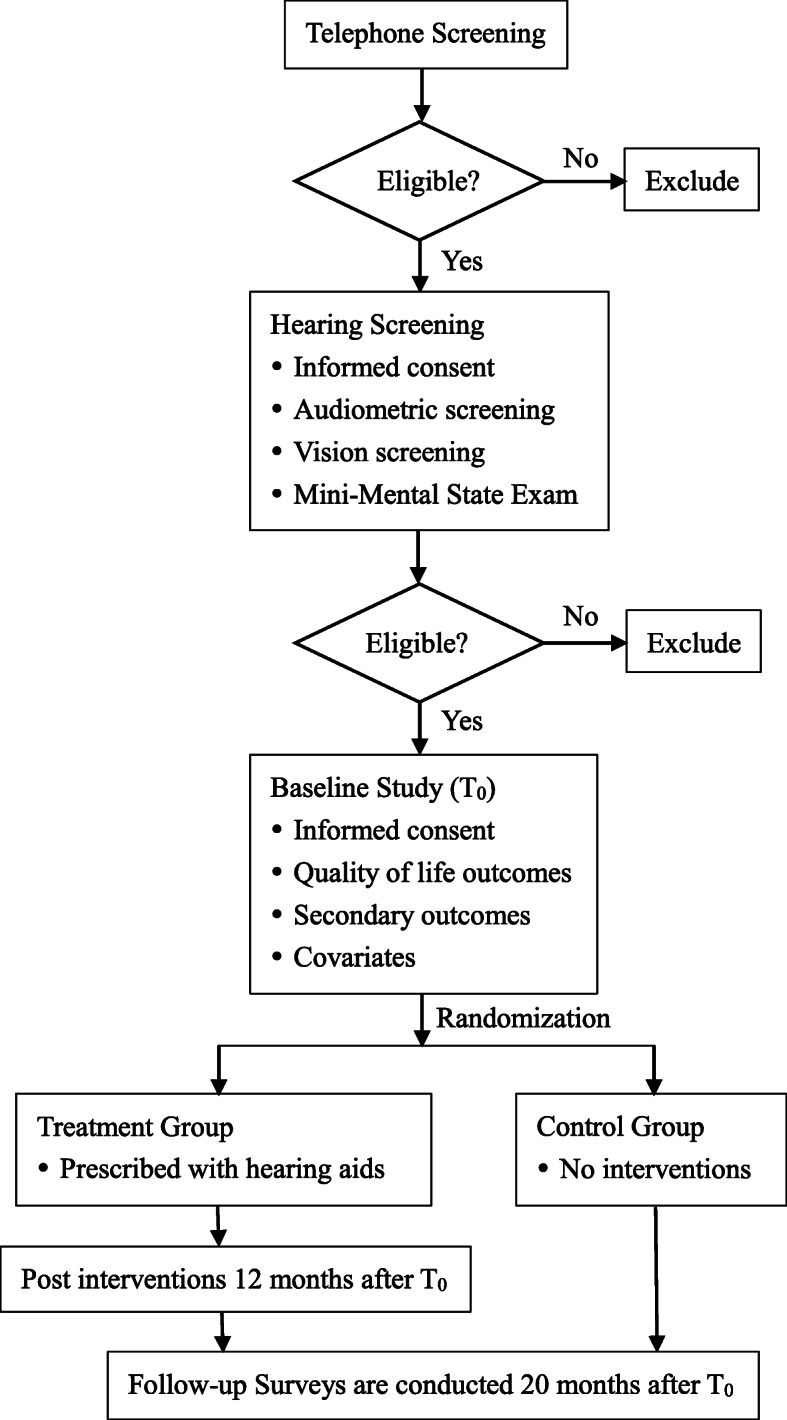
Participants screening and randomization. Note: Strata defined by severity of hearing loss

Our study is a randomized, controlled trial lasting 20 months with two parallel groups, the treatment group and the control group. According to a list provided by the Hearing Center of Linyi Disabled Persons’ Federation, which records all the hearing-disabled people in Linyi City, 464 people are randomly selected. Participants are prescreened by telephone and complete a hearing screening at baseline (T0). Those in the treatment group are prescribed with hearing aids and receive a post intervention of reinstruction in use of hearing aids 12 months after T0, while the control group receives no interventions. The follow-up surveys are scheduled 20 months after T0 to trace changes in health outcomes (Fig. 1).

### Eligibility criteria

Eligibility criteria are designed to identify community-dwelling adults with hearing loss who may possibly benefit from hearing rehabilitation (Table 1).

**Table 1:** Inclusion and exclusion criteria

Criteria	Description
Inclusion	• Age 16 years old and above
	• Diagnosed with some degree of hearing loss and are required to wear a hearing aid
	• Plans to stay in the geographic area for study duration
	• Community-dwelling
	• Fluent Chinese speaker
Exclusion	• Inability to read or write
	• Cognitive, mental, language, or movement disability diagnosis
	• Self-reported use of a hearing aid in the past 1 year
	• Unwilling to wear hearing aids on daily basis
	• Medical contraindication to use of hearing aids (e.g., draining ear)
	• Conductive hearing loss with air-bone gap > 15 dB in two or more contiguous frequencies in both ears that cannot be resolved

To be included in the study, each person must meet the following inclusion criteria: Participants are adults aged 16 years and above with untreated bilateral hearing loss and are required to wear a hearing aid by otologists. Participants are community-dwelling and will remain in the local area during the study period. They should be fluent Chinese speakers.

Exclusion criteria include inability to read or write, prior cognitive, mental, language or movement disability diagnosis, medical contraindication to hearing treatment (e.g., draining ear), untreatable conductive hearing loss (difference in air audiometry and bone audiometry (“air-bone gap”) > 15 dB in two or more contiguous frequencies in both ears that cannot be medically resolved), or unwillingness to regularly wear hearing aids.

### Study interventions

Participants in the hearing treatment group are prescribed with hearing aids at baseline, while the control group with no interventions. Reinstruction in use of hearing aids is prescribed during booster visits held 12 months and 20 months post-randomization. Unscheduled interim visits may also be sporadically required (e.g., hearing aid malfunction). Participants in the control group will not have access to hearing aids. They will be asked to attend twice for this project: baseline and follow-up 20 months later.

### Modifications

Study participation and hearing intervention are expected to have a low risk of adverse events. But the age of the participants may naturally lead to certain deleterious health outcomes. In case of any accidental injury, lack of efficacy, or withdrawal of participant consent during the trial, investigators can modify or discontinue the trial with the approval of the principal investigator (PI).

### Adherence

Adherence to the study intervention will be assessed at each study visit using questions designed to capture adherence in both the treatment and control groups. Strategies to promote adherence include:

- Individual assessment and instruction.
- Participants are asked to bring a communication partner with them to the study visits.
- Participants who miss a scheduled meeting are contacted by telephone by study staff to encourage continued participation and to evaluate and overcome barriers to participation.

For the treatment group, it additionally includes:

- Potential intervention benefits are structured given the participant’s level of hearing loss in order to ensure that participants’ expectations are reasonable and realistic.
- Participants are informed that they are allowed to keep the hearing aids for free if they complete all study visits.

### Outcomes measures

#### Primary outcome

The primary study outcome is the 20-month changes in quality of life from the 2019 baseline to the 2021 follow-up. Quality of life can be measured by standard tests of Short Form 12 Health Survey (SF-12) [29] and EuroQoL 5-Dimension (EQ-5D) [30].

#### Secondary outcomes

Key secondary outcomes include changes in sub-scores of SF-12 (physical and mental health) [29] and EQ-5D (mobility, self-care, usual activities, pain/discomfort, and anxiety/depression) [30]. Other secondary outcomes are health variables independently associated with hearing loss, including physical functioning (ADLs, IADLs) [31], chronic diseases, cognitive function (MMSE) [32], depressive symptoms (CES-D) [33], social support (LSNS) [34], hospitalizations, and falls, as well as the direct and indirect medical costs and loss of productivity in the study duration.

#### Hearing outcomes

Participants are required to receive pure tone audiometry at the thresholds of 0.5, 1, 2, and 4 kHz to derive accurate hearing data. Besides pure tone audiometry, standardized questionnaires like Hearing Handicap Inventory for the Elderly—Screening version (HHIE-S) [35], combined with participants’ self-reported hearing status, will be gathered to supplement the objective hearing data. For participants randomized to the hearing intervention group, audiologic outcomes to verify the intervention effects, such as the International Outcome Inventory for Hearing Aids (IOI-HA) [36], are gathered semiannually post-randomization.

#### Covariates

Sociodemographic factors such as age, gender, residency, family information, socioeconomic status, and other clinical factors are collected at baseline and the follow-up survey.

### Sample size

Power calculations showed that 404 (202 in each group) participants are required in each group to obtain 80% statistical power with a 5% significance level and to detect a 0.28-SD difference in the mean change from baseline in quality of life score to 20-month follow-up [37]. To account for drop-in (uptake of hearing aids in the control group) and drop-out (discontinuation of hearing aid use in the treatment group), 464 persons are included, of which 232 are in the intervention group and 232 in the control group.

### Recruitment

Our recruitment will last 1 month, from July 1 to 31, 2019. Adults with untreated hearing loss will be recruited according to the list of people with hearing loss provided by the hearing center of Linyi Disabled Persons’ Federation. Informed, written consent is obtained from all participants prior to participation.

### Randomization

Participants are randomized stratified by severity of hearing loss, so as to avoid uneven distribution of participants. A researcher who is not involved in data collection or analysis will use a random sequence generator (http://www.random.org) to allocate participants in a random sequence to the treatment or control group. In a separate room after completion of the baseline measurements, an independent researcher will tell participants to which group they have been allocated, thereby ensuring concealment of the identity and characteristics of participants.

### Blinding

Neither study participants nor researchers collecting outcome data can feasibly be blinded to randomization status. Precautions to minimize potential bias resulting from the lack of blinding include (1) blinding of participants to the study hypothesis, (2) use of standardized protocols for training of data collectors and assessment of study outcomes, and (3) masking of field working staff to block size, to avoid unintentional and possibly unconscious bias by study staff during data collection.

### Data collection

Standardized data forms or tablets are used to collect data with paper back up available in case of tablet failure. Participants will fill in all questionnaires in a separate room. To ensure that appropriate help and guidance can be given when needed, one of the researchers will present. Meanwhile, communication partners who communicate with participants on a daily or near-daily basis (e.g., spouse) are often a key to getting accurate results. Therefore, accompanying adults are also invited to join the study and contribute to the data.

To minimize data-entry errors, the questionnaires have inbuilt check-and-skip rules. And the questionnaires were tested on four participants beforehand, who found the questions understandable and possible to complete in 30–45 min. For participants who withdraw from the trial, any data collected up to the withdrawal date will be retained and included in the analyses. Data are collected at the local field prior to randomization (baseline T0), after 12 months (post intervention T1) and 20 months after baseline (follow-up T2). A SPIRIT flow diagram illustrates the data collection in the intervention group and control group (Table 2) [38].

**Table 2:** Schedule of enrollment, interventions, and assessments

Timepoint	Study period							
	Enrollment −T_1_ −30 to −1 day		Allocation T_0_ day 0		Post-allocation T_1_ 12 months		Close-out T_2_ 20 months	
	T ^a^	C ^b^	T ^a^	C ^b^	T ^a^	C ^b^	T ^a^	C ^b^
Enrollment								
Eligibility screen	×	×						
Informed consent	×	×						
Allocation			×	×				
Intervention			×		×		×	
Assessments								
Socio-demographic data	×	×	×	×			×	×
Hearing outcome								
Pure tone audiometry			×	×			×	×
HHIE-S score			×	×			×	×
Self-reported hearing			×	×			×	×
IOI-HA score					×		×	
Quality of life (primary outcome)								
SF-12 score			×	×			×	×
EQ-5D score			×	×			×	×
Secondary outcomes								
Sub-dimension of SF-12			×	×			×	×
Sub-dimension of EQ-5D			×	×			×	×
Physical functioning (ADLs)			×	×			×	×
Physical functioning (IADLs)			×	×			×	×
Chronic diseases			×	×			×	×
Cognitive function (MMSE)			×	×			×	×
Depression (CES-D)			×	×			×	×
Social support (LSNS)			×	×			×	×
Hospitalizations			×	×			×	×
Falls			×	×			×	×
Medical costs			×	×			×	×
Loss of productivity			×	×			×	×

*Abbreviations: T*, treatment group; *C*, control group; *HHIE-S*, Hearing Handicap for the Elderly-Screening Version; *IOI-HA*, The International Outcome Inventory for Hearing Aids; *SF-12*, Short Form 12 Health Survey; *EQ-5D*, EuroQoL 5-Dimension; *ADLs*, activities of daily living; *IADLS*, instrumental activities of daily living; *MMSE*, Mini-Mental State Examination; *CES-D*, Center for Epidemiologic Studies-Depression; *LSNS*, Lubben Social Network Scale

### Data management

The data entry is double-checked for errors or omissions by an investigator blinded to the participants’ group allocation. For data coding, some measures such as range checks in data values are conducted. Then data shall be filed and stored in categories, and have multiple backups on different disks or recording media.

### Statistical analysis

In the statistical analysis, intention-to-treat (ITT) principle is applied with conservative estimates of missing data [39]. Participants’ characteristics will be summarized using descriptive statistics (mean, standard deviation, frequency). Analysis of variance or *t* tests are performed to compare means; Mann-Whitney *U* tests are used to compare variables with non-normal distribution. Baseline data will be used to investigate the characteristics of participants who discontinue or deviate from the trial and/or intervention. The magnitude of changes over time across study groups will be examined by a multiple imputation analysis of covariance model, so as to evaluate the intervention effect.

In addition, some variables such as socioeconomic status and social support may potentially affect the utilization of hearing rehabilitative services, so the interaction between socioeconomic status or social support with the hearing intervention can be analyzed to gain further results. The cost-effectiveness analysis is based on the cost of the intervention, effects in improving health-related quality of life, decreasing inpatient and outpatient visits, and saving healthcare costs and recovering productivity. By these means, we aim to explore the most cost-effective intervention strategy to improve the quality of life for people with hearing loss.

### Data monitoring

The principal investigator (PI) is responsible for the quality and integrity of data collected. During the period of recruitment, interim analyses will be supplied in strict confidence, which may include analyses of data from other comparable trials. In the light of these interim analyses, the PI will advise if the intervention has been proved, or different from expected. Then the PI will decide whether or not to modify the trial. Unless this happens, however, study staff will remain ignorant of the interim results.

### Adverse events

In our study, adverse events will be collected and recorded after participants have provided consent and enrolled, until the end of the study. If a participant experiences an adverse event after the informed consent is signed (entry) but the participant has not started to receive study intervention, the event will be reported as not related to our intervention. An adverse event that meets the criteria for a serious adverse event (SAE) will be reported to the institutional review board (IRB). And study personnel will document the circumstances.

### Auditing and inspecting

PI will permit study-related monitoring, audits, and inspections by the IRB of all study related documents (e.g., source documents, regulatory documents, data collection instruments, study data). PI will ensure the capability for inspections of applicable study-related facilities.

### Patient and public involvement statements

This trial is carried out without patient or public involvement. Neither patients nor the public are involved in the development of the objective, design, or implementation of this trial. Patients will not be invited to develop patient-relevant outcomes or interpret the results, or to participate in the writing or editing of the final manuscript for readability or accuracy.

### Ethics/dissemination

#### Ethics approval and protocol amendments

The study was approved by Peking University’s Institutional Review Board (IRB00001052-19046). Any modifications to the protocol which may impact on the study, or the potential benefit and safety of the participants, including changes of study objectives, study design, participants, sample sizes, study procedures, or significant administrative aspects, will require a formal amendment to the protocol. Such amendment will be approved by the IRB prior to implementation.

#### Consent and confidentiality

Investigators will introduce the trial to participants in light of the information provided in the information sheets. Participants will then be able to have an informed discussion with the investigator. Investigators will obtain written consent from willing participants. For confidentiality, each participant will be given a unique identification number. Other identification information such as names, mobile phone numbers, and addresses will not be recorded in the same form as sensitive data. In case of any accidental injury during the trial, medical treatment and economic compensation will be provided according to the laws and regulations of China.

#### Dissemination

PI will be given access to the cleaned data sets. Results will be disseminated in the clinical and scientific communities and also to the population with hearing loss via peer-reviewed research publications both online and in print, conference and meeting presentations, posters, newsletter articles, website reports, and social media. Results will be reported according to the Consolidated Standards of Reporting Trials (CONSORT) guidelines [40]. Important protocol modifications will be reported when findings are disseminated.

## Discussion

The quality of life is associated with individuals’ physical health, psychological experience, and social relations [24]. It is relevant to multiple physical and mental diseases and social care systems [41]. Improvements in quality of life are important outcome measures for hearing treatment, both clinically and for researchers [42]. This paper describes a protocol for an RCT to evaluate the impact and cost-effectiveness of a community-based hearing aids intervention on quality of life among Chinese adults with untreated hearing loss.

This will be the first RCT accessing the efficacy of hearing aids intervention on quality of life among Chinese adults. Strengths of this study include the robust randomized controlled trial design, with randomization stratified by hearing loss severity to reduce contamination, the inclusion of follow-up measures, and the rigorous economic evaluation. This study will contribute to building the evidence base around the effectiveness of interventions to improve the well-being for Chinese adults and provide references for other countries.

We should note some weaknesses in the design of the trial. First, participants in the control group might also undergo changes in quality of life, which may conceivably be induced by questions about their health behaviors. It has been shown that participants’ behaviors can be increased or changed simply if questions are asked about their behaviors [43]. In our study, there is thus a chance that the effects of asking people about their behaviors will reduce any differences between the intervention and the control groups and thereby the possibility of finding a significant effect. The second limitation is that most data will be self-reported which are prone to bias. Therefore, we will design several corresponding questions in the questionnaires or conduct pre-test and post-test to avoid subjective bias. For example, the question about self-rated health is asked twice to verify the potential mistakes. Finally, this study is limited by being adaptable to the pragmatic realities of multi-country projects while maintaining scientific integrity, such as understanding how different health organizations work, how to observe ethics processes, how to get accurate translation of questionnaire scales, or how to gain permissions and copyright in different countries.

To conclude, whether hearing treatment and rehabilitation can delay quality of life decline in at-risk adults is lacking compelling evidence, but could have substantial clinical, social, and public health impacts for people with hearing loss. When completed in 2021, our study should provide definitive evidence of the cost-effectiveness of hearing aids treatment on quality of life in community-dwelling Chinese adults with hearing loss.

### Trial status

The current protocol is version 2, dated July 26, 2019. It took quite a few months to select journals and submit the protocol for review. Recruitment of patients began on July 1 and ended on July 31, 2019. The data collection ran until all participants completed the survey. Follow-up will be conducted after 20 months to test whether the effects of the intervention are still present. The study is expected to run until the end of March 2021. Until then, the intervention effects are unknown.

## Supplementary Information

**Additional file 1.** Trial Registration Data.

**Additional file 2.** Informed Consent Forms.

## Data Availability

Not applicable.

## Abbreviations

RCT: Randomized controlled trial
SF-36: Short Form 36 Health Survey
EQ-5D: EuroQoL 5-Dimension
PI: Principal investigator
SF-12: Short Form 12 Health Survey
ADLs: Activities of daily living
IADLs: Instrumental activities of daily living
MMSE: Mini-Mental State Examination
CES-D: Center for Epidemiologic Studies-Depression
LSNS: Lubben Social Network Scale
HHIE-S: Hearing Handicap for the Elderly-Screening Version
IOI-HA: The International Outcome Inventory for Hearing Aids
ITT: Intention-to-treat
SAE: Serious adverse event
IRB: Institutional review board
CONSORT: Consolidated Standards of Reporting Trials
SPIRIT: Standard protocol items: recommendation for interventional trials
