# Supplementary material for: Impact and cost-effectiveness evaluation of a community-based rehabilitation intervention on quality of life among Chinese adults with hearing loss: study protocol for a randomized controlled trial
Source: Trials. 2021 Apr 7;22:258. doi: 10.1186/s13063-021-05228-2 (PMC8028700; doi:10.1186/s13063-021-05228-2)
Supplement: Supplementary file 1 — Additional file 1. Trial Registration Data. [file 13063_2021_5228_MOESM1_ESM.docx]

**Additional File 2. Trial Registration Data**

| **Data category** | **Information** |
| --- | --- |
| Primary registry and trial identifying number | Chinese Clinical Trial Registry; ChiCTR1900024739 |
| Date of registration in primary registry | 26 July, 2019 |
| Source(s) of monetary or material support | National Natural Science Foundation of China;  Peking University's Start-up Fund |
| Primary sponsor | National Natural Science Foundation of China |
| Secondary sponsor(s) | Peking University's Start-up Fund |
| Contact for public queries | Xin Ye, PhD Candidate, School of Public Health, Beijing, China |
| Contact for scientific queries | Xin Ye, PhD Candidate, School of Public Health, Beijing, China |
| Public title | Impact and cost-effectiveness evaluation of community-based rehabilitation interventions on quality of life among older adults with hearing loss |
| Scientific title | Impact and cost-effectiveness evaluation of community-based rehabilitation interventions on quality of life among older adults with hearing loss |
| Countries of recruitment | China |
| Health condition(s) or problem(s) studied | Hearing loss |
| Intervention(s) | Treatment group: Prescribed with hearing aids |
|  | Control group: No interventions |
| Key inclusion and exclusion criteria | Inclusion criteria:  Adults aged 16 and above;  Diagnosed with some degree of hearing loss and are required to wear a hearing aid;  Plans to stay in the geographic area for study duration;  Community-dwelling;  Fluent Chinese speaker. |
|  | Exclusion criteria:  Inability to read or write;  Cognitive, mental, language or movement disability diagnosis;  Self-reported use of a hearing aid in the past 1 year;  Unwilling to wear hearing aids on daily basis;  Medical contraindication to use of hearing aids (e.g., draining ear);  Conductive hearing loss with air-bone gap >15 dB in two or more contiguous frequencies in both ears that cannot be resolved. |
| Study type | Interventional |
|  | Allocation: randomized; masking |
|  | Primary purpose: rehabilitation |
| Date of first enrolment | Aug. 2019 |
| Target sample size | 464 |
| Recruitment status | Recruiting |
| Primary outcome(s) | Quality of life |
| Key secondary outcomes | Sub-scores of the quality of life; physical functioning; chronic diseases; cognitive function; depressive symptoms. |
