# Supplementary material for: Impact and cost-effectiveness evaluation of a community-based rehabilitation intervention on quality of life among Chinese adults with hearing loss: study protocol for a randomized controlled trial
Source: Trials. 2021 Apr 7;22:258. doi: 10.1186/s13063-021-05228-2 (PMC8028700; doi:10.1186/s13063-021-05228-2)
Supplement: Supplementary file 2 — Additional file 2. Informed Consent Forms. [file 13063_2021_5228_MOESM2_ESM.docx]

**Additional File 3. Informed Consent Forms**

**Participant’s consent form**

- I have read the information letter. I had the opportunity to ask questions. If I had any questions, they were answered properly. I was given plenty of time to make a decision on participating in the study.
- I know the purpose of the research and how much time it will take me to participate in it.
- I give my full consent to the use of my data for the express purposes detailed in the information letter. I fully consent to the fact that all data will be stored for up to 5 years after completion of the research.
- I know that participation is voluntary. I know that I can decide at any given moment to withdraw this participation without stating a reason.
- I agree to participate in this research.

Name of participant:

Signature: Date : __ / __ / __
